# The Influence of Factors Such as Parenting Stress and Social Support on the State Anxiety in Parents of Special Needs Children During the COVID-19 Epidemic

**DOI:** 10.3389/fpsyg.2020.565393

**Published:** 2020-12-10

**Authors:** Jie Ren, Xingkai Li, Shudan Chen, Suiqing Chen, Yangang Nie

**Affiliations:** ^1^Department of Special Education, School of Education, Guangzhou University, Guangzhou, China; ^2^Department of Psychology, School of Education, Guangzhou University, Guangzhou, China; ^3^Department of Preschool Education, School of Education, Guangzhou University, Guangzhou, China; ^4^Department of Psychology and Research Center of Adolescent Psychology and Behavior, School of Education, Guangzhou University, Guangzhou, China

**Keywords:** parents of special needs children, state anxiety, COVID-19, parenting stress, social support

## Abstract

**Objectives:**

The study aims to investigate the state anxiety of parents of special needs children during the 2019 coronavirus disease (COVID-19) epidemic and the influence of parental stress, social support, and other related variables on the anxiety of parents.

**Methods:**

Bespoke questionnaires of children’s and parent’s mental and behavioral problems during the epidemic were used in the study. We also used the State Anxiety Inventory (S-AI), the Parenting Stress Index—Short Form-15 (PSI-SF-15), the NEO Five-Factor Inventory (NEO-FFI), and the Multidimensional Scale of Perceived Social Support (MSPSS). The data used in the study were pooled from an online survey of parents of special needs children and analyzed by one-way analysis of variance (ANOVA) and multiple linear regression.

**Results:**

Overall, 1,451 individuals were included, of which 402 were fathers (27.71%) and 1,049 were mothers (72.29%). ANOVA results showed that educational background, family monthly income, and type of their child’s disability made parents’ state anxiety significantly different. The results of multiple linear regression showed that during the epidemic, social support negatively predicted parents’ state anxiety (*B* = −0.15, *p* < 0.001), whereas parenting stress (*B* = 0.07, *p* = 0.001) and parental mental and behavioral problems (*B* = 0.37, *p* < 0.001) positively predicted parents’ state anxiety.

**Conclusions:**

During the outbreak of COVID-19, parents of special needs children suffered mental and behavioral problems, together with parenting stress and social support, which influenced their state anxiety. These findings can be used to develop relevant psychological interventions to improve the mental health of vulnerable groups during a pandemic like COVID-19.

## Introduction

The 2019 coronavirus disease (COVID-19) began to spread in China in January 2020. Travel restrictions were then imposed in most areas of the country. The Spring Festival holiday was extended, enterprises stopped, work was put on hold, and schools closed. People stayed at home and socially isolated in order to avoid being threatened by the virus (Wang et al., 2020). Some studies believe that for people staying at home, although it can effectively cut-off the spread of the virus, the isolation also harms people’s mental health, causing psychological problems, such as anxiety, insomnia, depression, and stress-related symptoms (Liu et al., 2020). Special needs children cover children with chronic conditions as well as children with disabilities ranging from mild to severe impairment (Caicedo, 2014). There are mainly three kinds of special education schools in China: schools for visually impaired students, schools for hearing impaired students, and schools for intellectual disability, including intellectual disabled and autistic students. In this study, we selected the parents of children with autism, intellectual disability, visual impairment, and hearing impairment as valid participants. During the epidemic period, parents of special needs children might face more hardships and exert more effort to take care of their children’s learning and living conditions than parents of typically developing children. Therefore, we should investigate the psychological situation of parents of special needs children to help them through this difficult period.

State anxiety is a form of anxiety that differs in each individual. It is a temporary, anxious emotional state or reaction triggered by a specific situation. Its intensity and volatility also change with time (Spielberger and Gorsuch, 1983). The study posits that the level of parental anxiety affects the physical and mental development of infants and children (Ramchandani et al., 2005). There is an epidemiological relationship between anxiety, depression, mental illness, negative life events, and the poor quality of a relationship with a partner (Pripp et al., 2010). A study of health care workers during the COVID-19 outbreak showed that a significant number of people reported symptoms of anxiety, especially for health care workers in areas, such as Wuhan, that were severely affected by the epidemic (Lai et al., 2020). There have been many studies reporting the anxiety of medical staff and ordinary people during the epidemic, but few studies have focused on vulnerable groups. Under the policy of suspension of work and school, schools and rehabilitation training institutions are closed, and special needs children stay at home. Normal school education, rehabilitation training, individualized interventions, and even treatment cannot be carried out, and some children inevitably experience behavior regression. Living at home also causes the child to have difficulty adapting and brings about more behavioral and emotional problems that need to be dealt with by the parents. Parents of special needs children have to undertake the tasks of child care, training, rehabilitation, and learning during the epidemic that poses a very big challenge for them. Exploring their anxiety under stress and the corresponding influencing factors will help to carry out targeted guidance and assistance.

Parenting stress is defined as a special type of stress that stems from the requirement to be a good parent. Parenting stress is caused by children’s needs and emotional conditions, as well as parents’ health characteristics, which determine the overall level of stress a parent can feel in their parenting role (Abidin, 1992). Behavioral problems of children and the parenting stress felt by parents are interrelated (Puff and Renk, 2014). A longitudinal study conducted by Neece and Baker (2008) showed that children’s behavioral problems are an effective predictor of parenting stress. Compared with typically developing children, special needs children, such as with intellectual disability (Chan and Lam, 2017) and autism (Kim et al., 2016), have more behavioral problems, which may bring more parenting stress to their parents. On the other hand, a study on parents of preschool children by Skreden et al. (2012) found that the parents’ anxiety was related to their parental stress. A study of parents of diabetic children also found that parental stress predicts their anxiety level. The higher the parenting stress, the higher the anxiety level (Streisand et al., 2008). It is reasonable to believe that parents of special needs children have to bear more parenting stress than usual during the epidemic because they have to take care of their children’s education and overall well-being, which eventually leads to an increase in the anxiety level of the parents. Therefore, investigating parenting stress during the epidemic and exploring its influence on their anxiety have special significance for helping them achieve good mental health during the epidemic.

Perceived social support refers to the emotional experience and satisfaction that individuals feel respected, supported, and understood in society (Norris and Kaniasty, 1996). Perceived social support affects people’s behavior and development through the psychological reality of the subjective perception of support and is more likely to show the beneficial function for individual mental health. Perceived social support serves as a buffer between stress and mental health. People can get the beneficial effects of reducing anxiety levels or solving problems from the support of family, friends, or neighbors (Zhou et al., 2013). A study conducted by Xiao et al. (2020) found that the anxiety of medical staff treating patients with COVID-19 infection was negatively correlated with their social support level from January to February 2020. The higher the level of social support, the lower the self-reported anxiety. During the epidemic, parents of special needs children may want to get help and support from others to solve their child care problems. This study sought to explore the influence of social support on anxiety of parents during the epidemic period, in order to provide parents with targeted social support and help them to reduce anxiety.

All in all, we conducted a social survey on the anxiety, parenting stress, and perceived social support under the COVID-19 epidemic to understand the anxiety of parents of special needs children and its related influencing factors. At the same time, we also investigated the mental and behavioral problems encountered by special needs children and parents during the epidemic. We want to provide more targeted theoretical basis for family support services and help special needs children and their parents have better mental health in their home life during the epidemic.

## Materials and Methods

### Participants and Procedures

The online survey targeted the parents of special needs children in Guangdong Province of China and was conducted from February 18 to February 22, 2020. Participants were sampled by stratified random sampling method and recruited through special education schools and special education agencies in Shenzhen, Guangzhou, Shaoguan, Dongguan, Foshan, Huizhou, and other 21 cities or regions. A total of 1,898 responses were received, of which 1,750 were valid, with a valid rate of 92.20%. Participants of the current study were 1,451 parents of children with autism, intellectual disability, visual impairment, and hearing impairment, of which 402 were male respondents (*M*_age_ = 42.79, *SD* = 5.58, *Range* = 18–60), accounting for 27.71%, and 1,049 were females (*M*_age_ = 39.63, *SD* = 5.31, *Range* = 18–60), accounting for 72.29%.

### Measures

#### Demographic Information and General Situation Under COVID-19

The demographic information of the study included gender, age, region, educational background, family monthly income, employment situation before the epidemic, current work situation, and other information of special needs children’s parents. Information about the child’s gender, age, type of special barriers, grade level, and caregivers was also collected.

#### Mental and Behavioral Problems of Children During COVID-19

Special needs children’s Mental and Behavioral Problems (CMBP) during the epidemic was a bespoke questionnaire, which was used to measure the problems of special needs children during home isolation. The questionnaire contained six questions, such as reluctance to wear masks, reluctance to wash hands, request to go out, sleep problems, eating problems, mood swings, etc. Each question was answered on a 4-point Likert scale, ranging from 1 (none) to 4 (many). In this study, the internal consistency reliability of the questionnaire was 0.76. A confirmatory factor analysis of the questionnaire showed that the model fits well (χ^2^/*df* = 4.89, *CFI* = 0.99, *TLI* = 0.98, *SRMR* = 0.02, *RMSEA* = 0.05), indicating that the questionnaire had good structural validity.

#### Mental and Behavioral Problems of Parents During COVID-19

The parents’ Mental and Behavioral Problems Questionnaire (PMBP) was a bespoke questionnaire, which was used to measure parents’ psychological and behavioral problems in the face of the epidemic. The questionnaire included 11 questions, such as “I couldn’t help to go to the hospital or repeatedly seek online consultations to confirm whether I have been infected with COVID-19” and “I couldn’t control my browsing information related to the epidemic (e.g., see WeChat moments, Weibo, WeChat group, etc.)”. Each question also used a 4-point Likert-type scoring method, ranging from 1 (inconsistent) to 4 (consistent). In this study, the internal consistency reliability of the questionnaire was 0.78. A confirmatory factor analysis of the questionnaire showed that the model fits well (χ^2^/*df* = 4.88, *CFI* = 0.96, *TLI* = 0.95, *SRMR* = 0.03, *RMSEA* = 0.05), indicating that the questionnaire has good structural validity.

#### Parenting Stress Index—Short Form-15

The Parenting Stress Index—Short Form-15 (PSI-SF-15) was a self-report questionnaire composed of 15 questions. It was revised by Luo et al. (2019) according to the Parenting Stress Index, which was used to measure the level of parenting stress. Each question was scored by a 5-point Likert scoring method, ranging from 1 (very much disagree) to 5 (very much agree). There were three subscales: (1) parental distress (PD): the higher the score, the greater the perceived parenting stress; (2) parent–child dysfunctional interaction (PCDI): the higher the score, the worse the parent–child relationship; and (3) difficult children (DC): the higher the score of difficult children, the more difficult the parents think their children are to care for. The combined score of each subscale was the total score of the scale. The higher the total score, the greater the parenting stress (Lee et al., 2016). PSI-SF-15 was a reliable and effective tool for evaluating parental pressure in China (Luo et al., 2019). In this study, the Cronbach’s α values of the three subscales of PD, PCDI, and DC were 0.84, 0.84, and 0.90, respectively, and the PSI-SF-15 was 0.92.

#### Multidimensional Scale of Perceived Social Support

The Multidimensional Scale of Perceived Social Support (MSPSS) was used to measure perceived social support from family, friends, and other important individuals (Zimet et al., 1988). There were 12 items in total, 4 items in each subscale, answered on a 7-point Likert scoring method, ranging from 1 (very low support) to 7 (very high support). The higher the total score of the 12 items, the better the level of social support felt by the individual. MSPSS showed good reliability and validity in the study of Chinese student samples (Zhang et al., 2016). In this study, the Cronbach’s α values of the three subscales of family, friends, and other important members were 0.91, 0.91, and 0.87, respectively, and the total scale reliability was 0.95.

#### NEO Five-Factor Inventory

Costa and McCrae (1989) obtained a scale of NEO Five-Factor Inventory (NEO-FFI) including 60 items based on NEO-PI, which was used to measure five personality structures, such as neuroticism, conscientiousness, extraversion, agreeableness, and openness. Considering that neuroticism in personality had been proven to be a powerful predictor of anxiety in previous studies (Moore et al., 2014; Xin et al., 2014), in order to exclude the influence of neuroticism, the neuroticism subscale was used in this study. The scale included 12 items, each with five levels, from “strongly opposed” to “very supportive.” In this study, the Cronbach’s α value of the neuroticism subscale was 0.81.

#### State Anxiety Inventory

The State Anxiety Inventory (S-AI) in the State-Trait Anxiety Inventory (STAI) compiled by Spielberger and Gorsuch (1983) was used to evaluate the state anxiety of special needs children’s parents under epidemic stress. S-AI was composed of 20 questions, which were graded 1–4: 1—none, 2—some, 3—moderate, and 4—very obvious. Studies conducted on Chinese samples confirm that S-AI had good reliability and validity (Cui et al., 2016). In this study, the Cronbach’s α value of the S-AI was 0.90.

### Statistical Analysis

IBM SPSS Statistics 25 and Mplus 8.3 were used for data statistical analysis. In this study, Mplus was mainly used for confirmatory factor analysis to determine whether the structural validity of the bespoke questionnaires is acceptable. And, SPSS was mainly used to do one-way analysis of variance (ANOVA), bivariate correlations, and multiple linear regression. First of all, descriptive analysis was carried out to explain the demographic characteristics of parents of special needs children and mental and behavioral problems of children and their parents. Secondly, a one-way ANOVA was carried out to compare the effects of gender, educational background, family monthly income, employment situation during the epidemic, and children’s types of disabilities on parents’ state anxiety. For the *post hoc* test, the Tukey test was used in the case of the homogeneous variance of each group, and the Games–Howell test was used in the case of heterogeneity of variance of each group. Finally, we used multiple linear regression to explore the influence of parenting stress, social support, and other variables on the state anxiety of parents. Considering the impact of additional factors, we controlled the parents’ gender, age, family monthly income, educational background, and neuroticism. All tests were performed at a two-tailed level, and *p* ≤ 0.05 was considered significant.

## Results

### Content Summary

A total of 1,451 parents completed the questionnaire. The parents’ gender, educational background, family monthly income, employment situation during the epidemic, types of children’s disabilities, and children’s grades are shown in Table 1. A total of 448 students (30.88%) were residential students, and 1,003 students (69.12%) were day students before the outbreak of COVID-19. Since the outbreak began, 1,304 (89.87%) parents and 1,420 (97.86%) students have been at home for more than 15 days. A total of 1,100 (75.81%) parents reported that their children were under their care, 308 (21.23%) reported that their children were under the care of their elders, and the rest reported that their children were under the care of others.

**TABLE 1:** Statistical hypothesis testing for the influencing factors (*N* = 1,451).

**Variables**	***N* (%)**	**State anxiety**		
		***M* ± *SD***	***F***	**Partial η^2^**
**Sex**			2.86	0.00
Female	1,049 (72.29)	45.76 ± 9.45		
Male	402 (27.71)	44.83 ± 9.10		
**Educational background**			7.04***	0.01
Under junior high school	617 (42.52)	45.87 ± 8.48		
Senior high school	421 (29.01)	46.46 ± 9.06		
College degree and above	413 (28.46)	43.96 ± 10.67		
**Family monthly income**			14.00***	0.02
Under 5,000 CNY	843 (58.10)	46.35 ± 9.18		
5,000–15,000 CNY	495 (33.98)	44.91 ± 9.42		
15,000 CNY and above	113 (7.79)	41.71 ± 9.39		
**Work situation during COVID-19**			9.92***	0.01
Unemployed	914 (62.99)	46.26 ± 8.97		
Working from the office	313 (21.57)	44.80 ± 9.90		
Working from home	224 (15.44)	43.35 ± 9.78		
**Type of disabilities**			3.09*	0.01
Autism	453 (31.22)	46.16 ± 9.96		
Intellectual disability	700 (48.24)	45.40 ± 8.97		
Hearing impairment	183 (12.61)	45.64 ± 9.04		
Visual impairment	115 (7.93)	43.22 ± 9.48		
**Child’s grade**			1.11	0.00
Preschool	97 (6.69)	46.57 ± 9.86		
Lower grade	555 (38.25)	45.65 ± 9.28		
High grade	438 (30.19)	45.62 ± 9.35		
Middle school	361 (24.88)	44.83 ± 9.36		

*p < 0.05; **p < 0.01; ***p < 0.001.

### The Impact of Differences in Demographic Information on State Anxiety

One-way ANOVA was used to analyze the characteristics of state anxiety of parents during the epidemic. The data were analyzed by Shapiro–Wilk test, and the results showed that the data of each group did not conform to the normal distribution (*p* < 0.001). Combined with the Q–Q plots, the data distribution result can be considered as a little negative skew distribution (Field, 2009). When the sample size is large (*n* > 50), one-way ANOVA can be performed (Ghasemi and Zahediasl, 2012). After Levene’s Test of Homogeneity of Variance, except for the educational background, the variance of data in each group was uniform (*p* > 0.05). For groups with different educational backgrounds, Welch’s ANOVA was used to determine whether there were differences in anxiety levels, and Games–Howell *post hoc* test was used for multiple comparisons.

The specific results of the different tests are shown in Table 1. According to Richardson (2011), when Partial η^2^ is 0.01–0.06, the effect is small. In the study, the above factors have less impact on parents’ state anxiety. The results showed that there was no significant difference in state anxiety of parents across gender (*F* = 2.86, *p* > 0.05) and grades of children (*F* = 1.11, *p* > 0.05).

Parents with different educational backgrounds had significantly different state anxiety results. The results showed that parents with a college education or above had significantly lower state anxiety scores than those who only reached senior high school (*M*_*d*_ = −2.49, 95% CI: −4.03, −1.00) and under junior high school education (*M*_*d*_ = −1.91, 95% CI: −3.32, −0.54). There was no significant difference in the scores of state anxiety between the parents whose education level was at the junior high school level or below and the senior high school group (*p* > 0.05).

Parents with varying family incomes have significant differences in their state anxiety results. The results showed that the parents whose family income was less than 5,000 CNY had significantly higher state anxiety score than the group earning 5,000–15,000 CNY (*M*_*d*_ = 1.44, 95% CI: 0.21, 2.67) and the group earning more than 15,000 CNY (*M*_*d*_ = 4.65, 95% CI: 2.46, 6.82). The score of state anxiety of the parents whose family income was 5,000–15,000 yuan was significantly higher than that of the group whose family income was more than 15,000 yuan (*M*_*d*_ = 3.20, 95% CI: 0.93, 5.47).

And, there was a significant difference in parents’ state anxiety when they had different working conditions during the COVID-19 epidemic. The results showed that parents who were unemployed during the epidemic had significantly higher state anxiety scores than parents who worked from home (*M*_*d*_ = 2.91, 95% CI: 1.25, 4.51) and parents who worked from the office (*M*_*d*_ = 1.47, 95% CI: 0.04, 2.87).

Finally, there were significant differences in the results of state anxiety among children with different types of disabilities. The results showed that the score of parents’ state anxiety was significantly higher in children with autism than in children with visual impairment (*M*_*d*_ = 2.95, 95% CI: 0.44, 5.46). There was no significant difference in the scores of parents’ state anxiety between the autistic children group and the intellectual and hearing-impaired children group (*p* > 0.05).

### Influencing Factors of Parents’ State Anxiety During the COVID-19 Epidemic

We examined the relationship between children’s mental and behavioral problems, parents’ mental and behavioral problems, perceived social support, parenting stress, neuroticism, and state anxiety during the COVID-19 epidemic. The average, standard deviation, range, and related analysis results of all measurements were shown in Table 2.

**TABLE 2:** Descriptive statistics and inter-correlations among the variables.

**Variables**	***M* (*SD*) *Range***	**1**	**2**	**3**	**4**	**5**	**6**
1. CMBP	8.40 (2.98)	1					
	6–24						
2. PMBP	22.21 (5.39)	0.19**	1				
	11–42						
3. Social support	56.85 (12.95)	−0.11**	−0.09**	1			
	12–84						
4. Parenting stress	34.65 (12.01)	0.36**	0.35**	−0.13**	1		
	15–75						
5. Neuroticism	32.69 (7.03)	0.27**	0.29**	−0.32**	0.50**	1	
	12–58						
6. State anxiety	45.49 (9.36)	0.19**	0.36**	−0.34**	0.35**	0.48**	1
	20–79						

**p < 0.01.

The study used state anxiety as the dependent variable, and the type of special needs children, mental and behavioral problems of children or parents during the epidemic, social support, and parenting stress were used as dependent variables to explore the influencing factors of parents’ state anxiety. We included parents’ gender, age, educational background, family monthly income, and neuroticism as control variables. The maximum variance inflation factor (VIF) was 3.78, indicating that there was no multicollinearity between independent variables. The final model was significant, *F* = 60.21, *p* < 0.001, and accounted for 33% of the total variance, *R*^2^ = 0.33.

The results were shown in Table 3. The regression analysis results showed that among the independent variables included in the model, parents’ mental and behavioral problems, perceived social support, and parental stress were statistically significant. During the epidemic, social support negatively predicted parents’ state anxiety (*B* = -0.15, *p* < 0.001), whereas parenting stress (*B* = 0.07, *p* = 0.001) and parental mental and behavioral problems (*B* = 0.37, *p* < 0.001) positively predicted parents’ state anxiety. That is, less social support combined with greater levels of parenting stress and parental mental and behavioral problems was significantly related to more parenting stress during the COVID-19 pandemic.

**TABLE 3:** Influencing factors of parents’ state anxiety during COVID-19.

**Predictor**	**Goodness of fit index**			**Unstandardized coefficients**		**β**	***t***	**95% CI for *B***	
	***R***	***R*^2^**	***F***	***B***	***SE***			**Low**	**Up**
Gender^*b*^	0.58	0.33	60.21***	−0.22	0.47	−0.01	−0.46	−1.14	0.7
Age^*b*^				0.02	0.04	0.01	0.65	−0.05	0.1
Education^*b*^				−0.17	0.19	−0.02	−0.93	−0.54	0.19
Family income^*b*^				−0.18	0.21	−0.02	−0.87	−0.59	0.23
Neuroticism^*b*^				0.41	0.04	0.31	11.70***	0.34	0.48
Visual impairment^*c*^									
Autism				0.42	0.83	0.02	0.51	−1.21	2.05
Hearing impairment				1.51	0.92	0.05	1.64	−0.3	3.32
Intellectual disability				0.87	0.78	0.05	1.11	−0.66	2.41
Social support				−0.15	0.02	−0.21	−8.99***	−0.18	−0.12
Parenting stress				0.07	0.02	0.09	3.27**	0.03	0.11
CMBP				0.09	0.08	0.03	1.13	−0.06	0.23
PMBP				0.37	0.04	0.21	8.94***	0.29	0.45

^*a*^Dependent variable: state anxiety.^*b*^Control variable: gender, age, education, family income, and neuroticism.^*c*^This is a reference variable.***p < 0.001, **p < 0.01.

## Discussion

The main purpose of this study is to investigate the state anxiety of parents of special needs children during the COVID-19 epidemic, as well as the influence of parenting stress, social support, and other factors on the status anxiety of parents. We hope to provide a theoretical basis and support for the effective intervention of improving parents’ mental health. The results confirmed the influence of factors, such as parenting stress, social support, and other factors, on state anxiety of parents under the COVID-19 epidemic.

### The Effect of the Basic Situation of Parents on Their State Anxiety

It was found that parents with different educational backgrounds, family monthly incomes, and current working conditions had significant differences in state anxiety. *Post hoc* test results found that parents with a college education or above had the lowest level of anxiety compared with the other groups. This may be because parents with higher academic qualifications are more likely to learn and master the corresponding skills needed to deal with their anxiety in order to avoid the adverse effects of excessive anxiety on themselves.

At the same time, we also found that parents with a monthly family income above 15,000 CNY had the lowest level of anxiety. This is also understandable because parents who have lower monthly household incomes will have more worries and anxieties about themselves and their families’ future economic situation. We also found that parents who worked at home during the epidemic had the lowest level of anxiety compared with the other groups. This may be because parents who still must go out to work during the epidemic are more worried about the safety and learning problems of the isolated children during the epidemic, making their anxiety level higher. However, those working at home can take care of their children so they are more likely to know more about their children’s situation and be relatively reassured.

Sola-Carmona et al. (2016) showed similar results for parents’ state anxiety of blind children: higher material well-being, job satisfaction, and family satisfaction were related to parents’ lower anxiety level. Indeed, compared with the parents of typically developing children, the parents of special needs children make more effort to take care of their children and even sacrifice their job opportunities. When the family’s socioeconomic level declines, parents’ anxiety will further increase, which will harm their mental health. Our research results indicate that relevant government departments should increase support for vulnerable groups, such as families of special needs children. We can help the families of special needs children get through this extraordinary period better by giving them financial subsidies and advocating home office work.

### The Effect of Parents’ Mental and Behavioral Problems on State Anxiety

The results showed that the mental and behavioral problems of parents of special needs children were the effective predictors of parents’ state anxiety during the epidemic period, whereas the predictive effect of children’s mental and behavioral problems was not significant. This highlights that we should focus on the mental health of parents during the outbreak. Facing the psychological problems caused by the epidemic, the National Health Commission of the People’s Republic of China held an emergency psychological crisis intervention. Various mental health associations and organizations have established expert groups to prepare guidelines and public health education articles/videos for mental health professionals and the public, as well as online mental health service personnel. Apart from this, mental health professionals and expert teams are stationed in designated isolation hospitals to provide on-site services (Li et al., 2020). Therefore, our teachers and schools can make full use of the resources provided by the government and relevant organizations to help parents alleviate their psychological and behavioral problems to reduce their anxiety.

### The Effect of Parenting Stress on State Anxiety During the Epidemic

Compared with the parents of typically developing children, more studies have confirmed that parents of special needs children have to bear more parenting stress, which is mainly due to the lack of professional support and the persistence of children’s problems among other reasons (Dabrowska and Pisula, 2010; Hayes and Watson, 2013). Therefore, during the epidemic, when special needs children are not able to go to school normally, parents may have to bear more parenting stress. Our research results also show that during the epidemic, parenting stress is an effective predictor of parental anxiety. Faced with this epidemic, parents need to spend more time and energy to take care of their children than usual. Especially for special needs children, parents are more likely to feel more pressure than usual. Special needs children may not be able to adapt to online education, and there are few ways for parents to prevent viral infections during the epidemic. This suggests that our teachers should be more patient in communicating with parents when conducting online education for special needs children, giving parents advice on children’s specific problems, and cooperating with parents to better carry out online education. The above measures will help parents alleviate parenting stress during the epidemic.

### The Effect of Social Support on State Anxiety During the Epidemic

The results of a study on parents of deaf children confirmed that parents’ perception of social support is an effective protective factor for their parenting stress (Åsberg et al., 2008). Therefore, helping parents improve their sense of social support also helps reduce their parenting stress. Previous studies have explored the relationship between anxiety levels and social support for parents of children with chronic kidney disease (Zengin et al., 2018) and parents of cancer children (Bayat et al., 2008). Our findings are consistent with their findings, confirming that social support is a protective factor for excessive anxiety. Support from family members, friends, and other people in the community helps to reduce the anxiety of parents.

In the face of the epidemic, the parents have limited power. All sectors of society need to help them find coping strategies together, to pass this difficult period more smoothly. Some studies provide suggestions for us to solve similar problems. A recent study confirmed the effect of reality therapy on perceived social support and state anxiety of parents of special needs children (Tumlu et al., 2017). The effect of such research deserves our attention. And, several studies have confirmed that mindfulness can make people more positively predict perceived social support (Kuhl and Boyraz, 2017; Sun et al., 2019). This may be because mindfulness enables individuals to focus on their current experience and realize the support they receive from social networks. Therefore, special schools could also carry out online psychological training specifically for parents to help them have a good level of mental health.

In conclusion, this study investigated the anxiety of parents of special needs children in China during the COVID-19 epidemic, as well as the influence of parenting stress, social support, and other factors on parents’ anxiety. The families with special needs children are vulnerable groups, and they will bear more psychological burden for taking care of their children themselves for such a long quarantine time. Therefore, the whole society should pay more attention to the parents of special needs children to help them get through this difficult period better.

## Data Availability Statement

The raw data supporting the conclusions of this article will be made available by the authors, without undue reservation.

## Ethics Statement

The studies involving human participants were reviewed and approved by The Ethics Review Committee (IRB) of Education School, Guangzhou University. The patients/participants provided their written informed consent to participate in this study. Written informed consent was obtained from the individual(s) for the publication of any potentially identifiable images or data included in this article.

## Author Contributions

JR, SQC, and YGN designed the study. JR, SQC, YGN, and SDC collected the data. XKL and JR analyzed the data. XKL, JR, and YGN wrote the manuscript. All authors read and approved the manuscript.

## Conflict of Interest

The authors declare that the research was conducted in the absence of any commercial or financial relationships that could be construed as a potential conflict of interest.
